# Propofol Sedation for ERCP Procedures: A Dilemna? Observations from an Anesthesia Perspective

**DOI:** 10.1155/2012/639190

**Published:** 2012-01-05

**Authors:** Davinder Garewal, Pallavi Waikar

**Affiliations:** Department of Anaesthesia, St. George's Hospital, London SW17OQT, UK

## Abstract

Propofol sedation for endoscopic retrograde cholangiopancreatography (ERCP) procedures is a popular current technique that has generated controversy in the medical field. Worldwide, both anesthetic and nonanesthetic personnel administer this form of sedation. Although the American and Canadian societies of gastroenterologists have endorsed the administration of propofol by nonanesthesia personnel, the US Food and Drug Administration (FDA) has not licensed its use in this manner. There is some evidence for the safe use of propofol by nonanesthetic personnel in patients undergoing endoscopy procedures, but there are few randomized trials addressing the safety and efficacy of propofol in patients undergoing ERCP procedures. A serious possible consequence of propofol sedation in patients is that it may result in rapid and unpredictable progression from deep sedation to general anesthesia, and skilled airway support may be required as a rescue measure. Potential complications following deep propofol sedation include hypoxemia and hypotension. Propofol sedation for ERCP procedures is an area of clinical practice where discussion and mutual cooperation between anesthesia and nonanesthesia personnel may enhance patient safety.

## 1. Introduction

Endoscopic retrograde cholangiopancreatography (ERCP) is an uncomfortable procedure that requires adequate sedation or general anaesthesia for successful completion. Currently, a substantial number of these interventions are performed worldwide. For instance, over 48,000 procedures are carried out per annum in the UK alone [1]. With an increasingly ageing population, the number of ERCP's performed globally is likely to increase and with that the demand for adequate sedation. The procedures are performed under conscious or deep sedation, and a wide variety of personnel may be involved in the administration of the sedation. The scope of this paper is to address some controversial areas regarding sedation for ERCP's and to review safety and efficacy aspects of sedation techniques with special reference to propofol usage.

## 2. Controversial Areas

Sedation represents a continuum of states of consciousness from mild sedation to general anaesthesia. The ASA has defined several levels of sedation from minimal through moderate sedation (conscious) to deep sedation after which general anaesthesia ensues [2]. Most cases of ERCP are performed either under conscious sedation, using midazolam with opioid (commonly Demerol) or deep sedation using propofol. Internationally, nonanesthesia use of propofol is in widespread use for endoscopies, and an impressive track record of its safety for these procedures has been built up. Almost half a million cases have been reported with a low incidence of problems [3]. Further support for the use of propofol by nonanesthesia personnel has come from the American and Canadian society of gastroenterologists, who have endorsed its use by these personnel [4, 5].

However, the administration of propofol by nonanesthesia personnel for sedation, although popular, is a contentious issue. In 2005, the American society of gastroenterologists petitioned the FDA to remove the requirement that “for general anaesthesia or monitored anesthesia care (MAC) sedation, Diprivan injectable emulsion should be administered only by persons trained in the administration of general anesthesia and not involved in the conduct of the surgical/diagnostic procedure.” However, this petition was ultimately denied in 2010 [6]. The consequences of this ruling will become evident in the future.

In countries such as the UK, gastroenterologists commonly perform ERCP under conscious sedation. However, both the Royal College of Anaesthetists (UK) and the American Society of Anesthesiologists consider that propofol sedation should ideally be administered by anesthesia personnel [7, 8].

Deep propofol sedation for patients undergoing ERCP procedures is preferred to conscious sedation by gastroenterologists at our institution, as they feel that patients often tolerate the procedure better. There appears to be some evidence to support this view [9, 10]. Laurie et al. in a retrospective chart review compared anaesthesia related sedation to conscious sedation for 732 ERCP procedures [9]. 17 patients failed to complete the procedure due to inadequate sedation in the conscious sedation group versus 3 patients in the anaesthesia related group. In addition, they found that the success rate of ERCP was higher in the anaesthesia related group (8.2% failure versus 19.2%) [9]. Penston prospectively reviewed 1550 ERCP procedures performed mainly under conscious sedation. He found that patient cooperation under conscious sedation (with Midazolam and Demerol) was not ideal in a significant number of patients, and this lack of cooperation made the performance of the procedure more difficult [10]. Our experience shows that approximately 9% of patients presenting for ERCP under propofol sedation are referred because the procedure could not be performed under conscious sedation.

An important consideration regarding sedation is that although levels of sedation have been defined, the actual level of sedation in patients may easily fluctuate, depending on the amount of drug used and sensitivity of the patient. For instance, a significant number of patients under conscious sedation may progress unintended to a level of deep sedation [11]. Deep sedation, as defined by the ASA, can require airway intervention, and spontaneous ventilation may be inadequate to maintain oxygenation and gas exchange. Propofol has a narrow therapeutic window, and a small increase in dosage may cause a patient to progress from deep sedation to a state of general anesthesia. This is an important reason why some authorities such as the Royal College of Anaesthetists in the UK maintain that patients undergoing deep sedation require the same level of care as those under general anesthesia [7]. This represents a further area of controversy, which is the use of deep sedation (inadvertent or deliberate) by nonanesthetic personnel.

## 3. Safety of ERCP under Sedation

Significant complications such as hypoxemia, hypotension, and aspiration are potential risks in patients undergoing ERCP procedures, and important factors that can modify the severity of these events include patients' ASA status, patients' hydration and oxygenation status, and monitoring techniques used during the procedure.

The majority of ERCP's are done under sedation with an open airway. Interestingly, aspiration has not often been reported as a problem, as patients are usually fasted and have empty stomachs. In our experience, small amounts of regurgitation with no significant sequelae are not uncommon in these patients. However, prolonged or difficult procedures may be associated with increased risks of regurgitation and aspiration, and this has been highlighted in a bulletin from the New Zealand College of Anaesthetists [12].

Two UK authorities, NCEPOD, and the British Society of Gastroenterologists have reviewed the safety of performing ERCP under conscious sedation [1, 13]. The 2004 retrospective review done by NCEPOD entitled “scoping your practice” identified a critical incident rate of 9% in patients undergoing ERCP procedures under conscious sedation and quoted a mortality rate of 2%. However, there were a relatively small number of patients reviewed, and the authors acknowledged that there may have been significant underreporting of some of the complications due to the retrospective nature of the survey. It was significant that many of the procedures done were considered to be inappropriate and that 77% of patients were ASA 3 and above. The survey performed by the BSG in 2005 included a larger group of patients and identified a procedure-related mortality of 0.4% (significantly less than reported by the NCEPOD report). Patients were also more appropriately selected for procedures, with only 12.7% being above ASA 3. 

Deenadayalu et al. conducted a worldwide safety review on patients undergoing endoscopic procedures under propofol sedation and found 3 deaths in 456,918 procedures [3]. However, the number of patients undergoing deep sedation is unclear. 

Linder et al. [14] analyzed 2113 ERCP cases performed under nurse administered midazolam and narcotic sedation or anaesthesia personnel administered deep propofol sedation. There was a low complication rate. There was one death, less than 1% of cases had to be converted to general anaesthesia, and approximately 6% of cases were deemed to require general anaesthesia electively for the procedures.

Coté et al. [15] prospectively studied 799 cases of patients undergoing endoscopy (ERCP, EUS, and small bowel enteroscopy) procedures under propofol sedation and found a hypoxemia rate of 12.8% and a hypotension rate of 0.8% during the procedure. There was a premature termination rate of 0.6%. Over 60% of patients had an ASA of 3 or above. Airway maneuvers were required in 14.4%, and ASA status 3 or higher and increased BMI were independent predictors of the need for airway intervention.

Only a limited number of randomized trials have addressed the issue of sedation for ERCP procedures. Four of these compared conscious sedation with midazolam and Demerol to propofol sedation [16–19]. In all of these trials, nonanesthesia personnel were involved in administrating the sedation. Two of the trials involved ASA 1–3 patients [17, 19], whilst the other two also included ASA 4 patients [16, 18]. There was no immediate mortality in any of the techniques. In one study 5 patients had to have the procedure terminated because of sedation-related problems [19], but in the other three trials, sedation seems to have been successful in all patients irrespective of the type of sedation used. None of the studies identified any significant difference in outcomes such as hypoxemia and hypotension (systolic < 90 mm Hg) between the two sedation techniques. However, two papers appear not to have administered oxygen during the procedure and report relatively high incidences of hypoxia using both techniques of sedation [17, 19].

## 4. Practical Aspects

Results from the limited number of studies performed comparing propofol sedation with conscious sedation for ERCP procedures have definitively indicated that patients who have received propofol for the procedure have a better recovery profile [16, 17].

Patients presenting for ERCP may often be elderly and infirm, and a significant proportion of them will be in the ASA 3 and 4 categories. Cardiorespiratory complications may occur more frequently, and the elderly may have depressed airway reflexes, making them more prone to aspiration. Common sense would suggest that in this group of patients extra care is taken to ensure adequate hydration and oxygenation before the procedure commences. Coté et al. [15] have confirmed that patients with an ASA category of 3 or higher required significantly more additional airway maneuvers during sedation for advanced endoscopy procedures (including ERCP).

## 5. Guidance from Major Anesthesia Organizations

The Royal College of Anaesthetists (UK) in conjunction with the British Society of Gastroenterology has issued guidance for patients undergoing propofol sedation for procedures such as ERCP [7]. The American Society of Anesthesiologists has also issued a guidance statement for the safe use of propofol in the context of sedation [8]. The specialist bodies' opinion is that propofol sedation requires specific training and skills for the following reasons.

- Propofol has potential to cause rapid and profound changes in sedative/anesthetic depth.
- Propofol has no specific antagonists.
- Propofol can have marked synergy with other drugs.

The guidance addresses the issues of personnel administering the sedation, patient selection, equipment and monitoring, staffing levels, and generic training. Briefly, the recommendation is that propofol sedation is ideally administered by dedicated and appropriately trained anesthesia personnel, but where this is not possible, dedicated and appropriately trained nonanesthesia personnel can take on this role, provided certain conditions are met. Patients should be adequately screened as to their suitability for this form of sedation as certain groups of patients (e.g., those with morbid obesity or severe cardiac or respiratory disease) are more at risk of developing complications during the sedation. For these group of patients, full general anesthesia with controlled ventilation should be planned. There should be a minimum level of cardiorespiratory monitoring used in patients undergoing propofol sedation, and this should include pulse oximetry and continuous capnography in addition to EKG and noninvasive blood pressure. All patients should receive continuous oxygen from the start of the procedure till the recovery period.

## 6. Propofol Sedation Experience at St. George's Hospital, UK

At our institution, propofol sedation for ERCP procedures is administered by consultant anesthesiologists (referred to as anaesthetists in UK). We follow a standard protocol for the administration of propofol, and all patients receive supplemental oxygen during the procedure. We monitor noninvasive blood pressure, EKG, end tidal carbon dioxide (ETCO2), and pulse oximeter oxygen saturation during the procedure as recommended by the Royal College of Anaesthetists and American Society of Anesthesiologists. The ETCO2 monitoring is facilitated by means of a modified mouthguard (manufactured by Pennine), which is capable of simultaneously delivering oxygen and sampling carbon dioxide from the patient, whilst facilitating the insertion of the endoscope [20]. The capnograph trace from this device is highly reliable, and we find it invaluable for maintaining the appropriate sedation level of the patient and detecting respiratory depression or obstruction. Qadeer et al. have demonstrated that the rate of hypoxemia can be reduced during ERCP procedures by incorporating capnograph monitoring [21].

Our experience with this technique in approximately 150 ASA1-3 patients undergoing ERCP with propofol sedation suggests that a low complication rate can be achieved. Results of our as yet unpublished prospective audit data demonstrate a transient hypoxemia rate of approximately 2%, and a transient hypotension rate (<90 mm Hg) of 5%. Sedation was successful in all patients. 9% of patients presenting for the ERCP procedure under propofol sedation had previously not tolerated the procedure under conscious sedation. 2 (1.3%) patients received elective intubation with general anesthesia for the ERCP procedure. Our indications for intubation include obesity, severe respiratory disease, anticipated prolonged procedure, and history of severe regurgitation. No patients had to be intubated as an emergency.

## 7. Development of Proficiency in Propofol Sedation for Nonanesthesia Personnel

We recommend following the guidance provided by the Royal College of Anaesthetists and American Society of Anesthesiologists regarding use of propofol for sedation. We also recognize that it may not be feasible for anesthesia personnel to administer propofol sedation for patients undergoing ERCP procedures in many instances. In these situations, we believe that it is acceptable for nonanesthesia personnel to administer propofol to patients undergoing ERCP procedures, provided that they have been adequately trained and certain conditions are met. A suggested training curriculum for nonanesthesia personnel wanting to learn the technique of propofol sedation has been comprehensively covered by major American gastroenterology bodies under the components of didactic training, airway workshops, simulation training, and preceptorship [4], and we concur with these. These conditions we suggest are outlined below.

- The practitioner should be a dedicated and appropriately trained person, with adequate life support and airway rescue skills.
- He/she should be regularly involved in administration of propofol sedation for a sufficient number of patients undergoing ERCP procedures.
- A period of preceptorship with an anesthesiologist experienced in propofol sedation for endoscopy procedures should be a core part of training.
- Preceptorship should be part of an ongoing process, with regular updates and refresher sessions.
- Patients should be screened for suitability, and not have any risk factors suggesting development of complications under deep sedation (e.g., obesity, severe cardiorespiratory disease, and severe gastroesophageal reflux).
- The procedure should not anticipated to be unduly difficult or prolonged.
- The procedures should be performed in a center where immediate anesthesiology assistance is available.
- Recommended monitoring, including capnography should be performed on all patients.

We consider that there is a learning curve involved with acquiring the subset of technical and decision-making skills necessary to become proficient at administering propofol for sedation. In our opinion, these are best learnt over a period of time under the supervision of an anesthesiologist experienced in this field. In this context, it might be beneficial to consider use of Bispectral index monitoring (BIS) early on in the preceptorship, as this might facilitate objective assessment of level of sedation during the procedure [22]. The practitioner may also want to incorporate this into his/her practice after the period of training. We suggest that adequate proficiency could be achieved after administration of propofol for 100 patients requiring ERCP (under supervision) although we accept that this number is open to discussion. Some of the skills that we feel are important to acquire are listed below:

- familiarity with administration of protocol following a set protocol,
- recognition and treatment of common problems such as respiratory obstruction,
- deciding which patients would be best suited for elective full general anesthesia,
- anticipating when to abort the procedure so as to prevent escalation of complications,
- anticipating when to call for assistance from anesthesiology.

## 8. Cost Issues

There is no doubt that patients undergoing ERCP procedures under propofol sedation have a much improved recovery profile (both immediately and at 24 hours) than patients who have undergone the procedure under midazolam/meperidine conscious sedation [17]. The big question is who administers the sedation? If anesthesia personnel do so, then the cost of the procedure increases significantly, and gastroenterology societies and health insurance companies in the USA have taken the position that this is only warranted in high-risk patients [23]. Nurse administered propofol sedation is an alternative that would be cost effective but is one that may be opposed by anesthesiologists unless there is appropriate supervision and support. It also may not be a global solution, as legislative considerations may enter into the decision, depending on the country concerned.

## 9. Conclusion

Sedation for ERCP procedures is a challenging area for clinicians, where there is an overlap between anesthetic and nonanesthetic practice. Propofol can be administered relatively safely for endoscopic procedures by nonanesthetic personnel [3]. However, it is difficult to envisage a future without significant anesthesiology involvement in this controversial area. Cooperation and discussion between gastroenterologists and anesthesiologists may pave the way to a realizable solution. The final outcome will undoubtedly be determined by a mixture of financial, political, and scientific debate and may well differ internationally.
